# Inhibitors of inflammation and endogenous surfactant pool size as modulators of lung injury with initiation of ventilation in preterm sheep

**DOI:** 10.1186/1465-9921-11-151

**Published:** 2010-10-29

**Authors:** Noah H Hillman, Suhas G Kallapur, J Jane Pillow, Ilias Nitsos, Graeme R Polglase, Machiko Ikegami, Alan H Jobe

**Affiliations:** 1 Cincinnati Children's Hospital Medical Center, Division of Pulmonary Biology, Cincinnati, OH, USA 45236; 2 School of Women's and Infants' Health, The University of Western Australia, Perth, WA, Australia 6009

## Abstract

**Background:**

Increased pro-inflammatory cytokines in tracheal aspirates correlate with the development of BPD in preterm infants. Ventilation of preterm lambs increases pro-inflammatory cytokines and causes lung inflammation.

**Objective:**

We tested the hypothesis that selective inhibitors of pro-inflammatory signaling would decrease lung inflammation induced by ventilation in preterm newborn lambs. We also examined if the variability in injury response was explained by variations in the endogenous surfactant pool size.

**Methods:**

Date-mated preterm lambs (n = 28) were operatively delivered and mechanically ventilated to cause lung injury (tidal volume escalation to 15 mL/kg by 15 min at age). The lambs then were ventilated with 8 mL/kg tidal volume for 1 h 45 min. Groups of animals randomly received specific inhibitors for IL-8, IL-1, or NF-κB. Unventilated lambs (n = 7) were the controls. Bronchoalveolar lavage fluid (BALF) and lung samples were used to quantify inflammation. Saturated phosphatidylcholine (Sat PC) was measured in BALF fluid and the data were stratified based on a level of 5 μmol/kg (~8 mg/kg surfactant).

**Results:**

The inhibitors did not decrease the cytokine levels or inflammatory response. The inflammation increased as Sat PC pool size in BALF decreased. Ventilated lambs with a Sat PC level > 5 μmol/kg had significantly decreased markers of injury and lung inflammation compared with those lambs with < 5 μmol/kg.

**Conclusion:**

Lung injury caused by high tidal volumes at birth were decreased when endogenous surfactant pool sizes were larger. Attempts to decrease inflammation by blocking IL-8, IL-1 or NF-κB were unsuccessful.

## Introduction

Ventilation of preterm newborn lambs initiates inflammation in the lungs [1,2]. Like preterm sheep, ventilated very low birth weight (VLBW) infants have increased concentrations of the pro-inflammatory cytokines IL-8, IL-1β, IL-6, and MCP-1 in tracheal aspirates and these increased levels correlate with an increased risk of bronchopulmonary dysplasia (BPD) [3-5]. Ventilation of preterm infants with moderate respiratory distress increased plasma levels of IL-1β, IL-8 and TNF-α and decreased levels of the anti-inflammatory cytokine IL-10 [6]. Surfactant is the major variable determining the compliance of the preterm lung [7], and surfactant treatment will decrease lung injury [8]. However the initiation of ventilation at birth is a unique situation because the airways initially are airless and fluid filled, and without labor, little surfactant will have been secreted into the fetal lung fluid. The initial ventilation of the preterm lung will occur before much of the endogenous surfactant is secreted [9], potentially increasing the risk of injury in a lung that might be protected by surfactant. The initiation of ventilation at birth stretches the airways and triggers early growth response protein 1 (Egr-1) activation [10]. The pro-inflammatory cascade from Egr-1 signals through NF-κB to increase cytokines and chemokines [1,10].

At a given preterm gestational age, infants have variable lung maturation because of the abnormalities associated with the preterm delivery. An argument for allowing the infant to transition to air breathing with continuous positive airway pressure (CPAP) is that uncontrolled high tidal volume ventilation can be avoided [11]. However, many infants will require ventilation to achieve respiratory transition [12]. The variability in lung function in experimental animals at delivery is less than in humans because the pregnancies are normal and the deliveries are carefully controlled. However, in sheep fetal lung maturation is rapidly changing between 128 and 136 days gestation [13]. We used a standardized 15 min escalating tidal volume injury maneuver in preterm sheep delivered at 133-134 d gestation to test if inhibitors of IL-8, IL-1, or NF-κB would decrease injury responses. We used well described early response genes (HSP70, Egr-1) and acute phase cytokines (IL-1β, IL-6, IL-8, MCP-1), as well as inflammatory cells, to quantify the lung injury. We also evaluated the endogenous surfactant pool size to test how this variability modulated the standardized stretch injury.

## Methods

The investigations were approved by the Animal Ethics Committees of the University of Western Australia and Cincinnati Children's Hospital Medical Center.

### Ventilation protocol

Ewes at 133 d to 134 d gestation were anesthetized prior to operative delivery of lambs [2]. Following externalization of the head, an endotracheal tube was secured surgically [14]. After delivery, lambs were weighed and ventilated with heated and humidified gas with FiO_2 _of 0.4, rate 40 breaths/min, and inspiration time of 0.7 sec (Bournes BP200) without surfactant treatment. Lambs received ventilation without PEEP and with tidal volume (V_T_) targets of 8-10 mL/kg at 5 min, 12 mL/kg at 10 min, and 15 mL/kg by 15 min, followed by 1 h 45 min ventilation with a PEEP 5 cmH_2_0 and a target PaCO_2 _at 50-60 mmHg. The upper limits were 40 cmH_2_O for PIP and 10 mL/kg for V_T_. V_T _values were measured continuously with Florian Respiratory Monitors (Acutronic Medical Systems, Switzerland). FiO_2 _was adjusted to maintain an oxyhemoglobin saturation between 88-95%. The ventilated lambs had umbilical arterial and venous catheters placed, and were anesthetized with Remifentanil and Propofol [14]. The animals received a FiO_2 _of 1.0 for 3 minutes prior to receiving a lethal dose of intravenous pentobarbital (100 mg/kg) at 2 h after birth. Unventilated controls were euthanized prior to delivery.

### Treatment with inhibitors

Lambs were randomized to selected inhibitors of inflammation (n = 7 animals/group) given prior to delivery and prior to initiating high V_T _ventilation. Lambs were randomized to: 1) No inhibitor - received the *V*_T _injury maneuver followed by ventilation only, 2) a NF-κB Inhibitor: Parthenolide 5 mg IV and 5 mg given intratracheally, mixed with the fetal lung fluid (Sigma, St. Louis, MO), 3) an IL-8 inhibitor: nicotinanilide thioglycolate methyl ester (NTME) 10 mg IV (Synthrix Biosystems, Auburn, WA), 4) the IL-1 receptor antagonist: Anikinra at doses of 100 mg IV and 100 mg intratracheally (Amgen, Inc., Thousand Oaks, CA), or 5) unventilated controls. Doses and routes of administration for this pilot study were determined from prior experiments [15-17].

### Lung Processing and Analysis

Bronchoalveolar lavage fluid (BALF) of the left lung was used to determine total protein content [18], saturated phosphatidylcholine (Sat PC) and differential cell counts after cytospins [19]. Sat PC was recovered after treatment of organic solvet extracts of the BALF with osmium tetroxide by alumina column chromatography and quantified by phosphorus assay [20]. Tissue from the lung were snap frozen. Total RNA was isolated using a modified Chomzynski method, and 10 μg of total RNA was used for IL-1β, IL-6, and IL-8 RNAse protection assays [21,22]. The right upper lobe was inflation fixed at 30 cmH_2_0 with 10% formalin [23], and tissue sections were used for injury scores [1]. MCP-1 protein from BALF was analyzed by sandwich ELISA using anti-ovine MCP-1 antibodies [24]. Immunostaining protocols used paraffin sections (5 μm, transverse) of formalin fixed tissues with anti-human Egr-1 1:250 dilution (Santa Cruz, USA) or anti-ovine MCP-1 1:300 (internally produced) [1,2]. For HSP70 mRNA identification *in situ*, digoxigenin-labeled riboprobes were generated (Roche, USA) and developed per protocol [1].

### Statistics

All values are expressed as means ± SEM. Comparisons between intervention groups were made with two-tailed Mann-Whitney nonparametric tests, Welch t-tests, or ANOVA where appropriate. Significance was accepted at p < 0.05.

## Results

**Figure 1:**
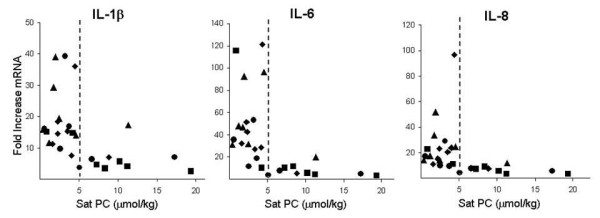
**Relationship of pro-inflammatory cytokine mRNA and Sat PC in BALF**. Scatter plots for cytokines based on Sat PC levels in BALF of individual lambs show decreased injury in lambs with Sat PC level > 5 μmol/kg. Cytokine mRNA levels were determined by RNase protection assays with L32 as an internal loading control. Values are expressed as fold increase of mRNA over unventilated controls, whose level was set to equal 1. ◆ No inhibitor ■ IL-1 inhibitor, ▲ NF-κB inhibitor ● IL-8 inhibitor.

All 28 lambs survived the 2 h ventilation period. There were no differences between cord blood gas measurements, birth weights, or gender between the groups. All animals achieved the V_T _goal of 15 mL/kg by 15 min and animals had similar V_T _ventilation throughout the 2 h study (Table 1). All ventilated lambs had increased BAL inflammatory cells, total protein, and MCP-1 protein compared with unventilated controls. There were no differences in the cytokine mRNA levels for IL-1β, IL-6 or IL-8 between the untreated or the inhibitor treatment groups. The lambs at this gestational age had large differences in surfactant pool size as measured by Sat PC in the BALF. The wide variances for cytokine mRNA within each treatment group correlated with the variation of Sat PC pool sizes (Figure 1). When analyzed in this way, the inhibitors had no effect on the injury within the limits of group sizes of 7 animals.

**Table 1:** Animals grouped by inhibitor treatments

	N	BW	V_T_ 15 min	V_T_ 2 h	BALF Protein	BALF Inflammatory cells	BALF MCP-1	IL-1β mRNA	IL-6 mRNA	IL-8 mRNA
			**mL/kg**		**mg/kg**	**x10**^**6**^**/kg**	**ng/mL**	**fold increase**		

Controls	7	3.2 ± 0.1	None		23 ± 6	1.2 ± 1.0	0.6 ± 0.6	1 ± 0.2	1 ± 0.2	1 ± 0.1

No Inhibitor	7	3.5 ± 0.2	15.7 ± 0.5	8.6 ± 0.8	82 ± 11*	81 ± 23*	28 ± 7*	16 ± 4*	43 ± 14*	28 ± 12*

NF-κB Inhbitor	7	3.6 ± 0.1	16.2 ± 1.1	9.3 ± 0.4	92 ± 18*	101 ± 34*	44 ± 10*	21 ± 4*	53 ± 11*	24 ± 5*

IL-8 Inhibitor	7	3.3 ± 0.1	15.5 ± 1.4	10.9 ± 0.9	72 ± 11*	63 ± 29*	32 ± 13*	14 ± 5*	20 ± 7*	12 ± 3*

IL-1 Inhibitor	7	3.6 ± 0.2	16.4 ± 0.7	9.6 ± 0.7	72 ± 18*	33 ± 11*	13 ± 7*	7 ± 2*	23 ± 15*	9 ± 3*

BALF = bronchoalveolar lavage fluid, BW = birth weight * p < 0.05 vs Controls.

**Figure 2:**
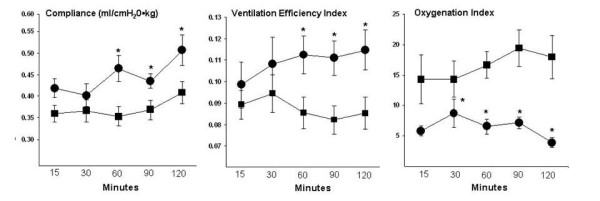
**Compliance, ventilation efficiency index, and oxygenation**. Lambs with Sat PC < 5 μmol/kg had decreased dynamic compliance values for the last hour of ventilation. Ventilation efficiency index decreased and oxygenation index increased in lambs with Sat PC < 5 μmol/kg, indicating decreased gas exchange over time. Oxygenation index at 2 h was measured on an FiO_2 _of 1.0. Compliance = V_T _/pressure, Ventilation Efficiency Index = 3800/(PIP•rate•PaCO_2_). Oxgenation index = (FiO_2_•Mean Airway pressure)/PaO_2. _● Sat PC > 5 μmol/kg ■ Sat PC < 5 μmol/kg * p < 0.05 vs Sat PC < 5 μmol/kg.

Lambs then were stratified into two groups based on the graphical presentations noted in Figure 1 into those with Sat PC levels < 5 μmol/kg in BAL (n = 18) and those with levels > 5 μmol/kg in BAL (n = 10). Unventilated control lambs had a Sat PC level of 2.0 ± 0.5 μmol/kg. All lambs had less Sat PC than the normal levels of approximately 65 μmol/kg reported for term lambs [25]. There were no differences in birth weight, gestational age, or gender ratio between lambs with Sat PC values greater or less than 5 μmol/kg (Table 2). The differences in Sat PC measured at 2 h did not significantly change the dynamic compliance during the 15 min V_T _injury maneuver (Table 2). Although no significant differences in V_T _or compliance were apparent during the 15 min stretch injury or at 30 min, lambs with Sat PC levels > 5 μmol/kg had improved ventilation, oxygenation, and compliances when ventilated with lower V_T _for the last hour of mechanical ventilation (Figure 2). Lambs with Sat PC < 5 μmol/kg required a FiO_2 _between 0.40 to 0.53 to maintain oxygen saturations, whereas the lambs with higher Sat PC levels required a FiO_2 _of only 0.25 to 0.39.

**Table 2:** Saturated phosphatidylcholine levels and indices of injury

	Sat PC < 5 (n = 18)	Sat PC > 5 (n = 10)	p value
**Description of Animals**			

Birth weight (kg)	3.5 ± 0.1	3.5 ± 0.1	= 1

Gender (male:female)	10:8	6:4	= 1

Gestational Age (days)	133.1	133.1	= 1

BAL Sat PC (μmol/kg)	2.5 ± 0.3	10.6 ± 1.4*	< 0.001

**V_T _and Compliance**			

V_T_/kg at 5 min (mL/kg)	10.2 ± 0.6	10.8 ± 0.7	= 1

V_T_/kg at 10 min (mL/kg)	14.4 ± 0.4	14.3 ± 0.5	= 1

V_T_/kg at 15 min (mL/kg)	15.6 ± 0.6	16.5 ± 0.5	= 1

Compliance 5 min (mL/cmH_2_0/kg)	0.28 ± 0.02	0.31 ± 0.03	= 0.50

Compliance 10 min (mL/cmH_2_0/kg)	0.34 ± 0.01	0.39 ± 0.02	= 0.11

Compliance 15 min (mL/cmH_2_0/kg)	0.36 ± 0.02	0.42 ± 0.02	= 0.06

**BAL Fluid**			

Protein (mg/kg)	94.4 ± 9.0	52.4 ± 7.8	< 0.002

Neutrophils (x10^6^/kg)	74.2 ± 1.8	16.2 ± 9.6	< 0.01

Monocytes (x10^6^/kg)	12.3 ± 2.9	8.9 ± 1.3	= 0.49

MCP-1 protein (ng/ml)	42.1 ± 5.6	7.2 ± 3.2	< 0.0001

**Lung Tissue **(mRNA fold increase)			

IL-1β	19.2 ± 2.3	6.2 ± 1.3	< 0.0001

IL-6	49.9 ± 8.0	7.2 ± 1.8	< 0.001

IL-8	24.8 ± 4.8	6.7 ± 0.8	< 0.0001

**Injury Scores**			

(Out of 8 total points)	4.8 ± 0.2	3.0 ± 0.5	= 0.02

V_T _= tidal volume, BAL = bronchoalveolar lavage fluid, Sat PC = saturated phosphatidylchloline.

BW = birth weight, Compliance = (VT/kg)/(PIP-PEEP) * p < 0.05 vs Sat PC < 5.

**Figure 3:**
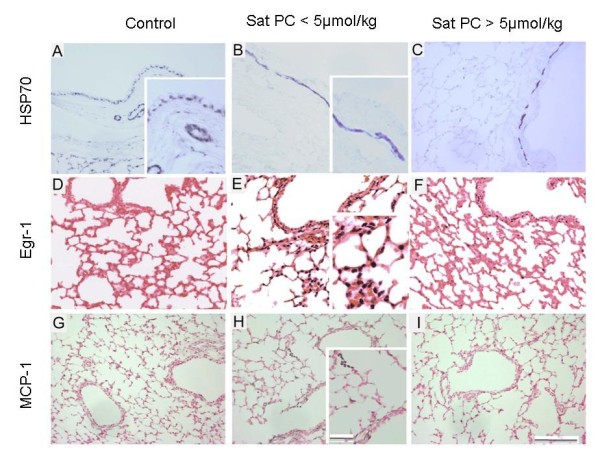
**Localization of HSP70 mRNA, Egr-1 protein and MCP-1 protein**. HSP70 *in situ *localization demonstrates signal in bronchial epithelium in controls (A), which is lost in ventilate lambs (B, C). Induction of HSP70 mRNA in smooth muscle surrounding airways is seen in lambs with < 5 μmol/kg Sat PC (B) and lambs with >5 μmol/kg (C). Egr-1 protein is not in airways of control lambs (D) but Egr-1 protein staining is qualitatively increased in lambs ventilated with < 5 μmol/kg Sat PC (E) verses lambs with > 5 μmol/kg (F). Compared to MCP-1 in controls (G), MCP-1 protein is localized to the mesenchyme surrounding the medium sized airways and to the inflammatory cells in lambs ventilated with < 5 μmol/kg Sat PC (H) with minimal staining in animals with > 5 μmol/kg (I). Scale bar = 50 μm, insert 25 μm.

All markers of lung injury were higher in animals with Sat PC levels below 5 μmol/kg in BALF than in the animals with higher Sat PC levels (Table 2). All ventilated animals had airway injury with sloughing of epithelium and inflammatory cells. Injury scoring between groups was higher in low Sat PC animals (Table 2). *In situ *localization of HSP70 mRNA demonstrated loss of the mRNA from bronchial epithelial cells in the ventilated animals compared with the unventilated controls and increased HSP70 mRNA in the smooth muscle surrounding the airways with ventilation (Figure 3A-C). There were no differences in HSP70 mRNA expression, representing similar airway over-expansion during ventilation. Early growth response protein 1 (Egr-1) surrounded the mesenchyme of the larger airways and was expressed in the medium sized conducting airways (Figure 3D-F). Egr-1 staining tended to be higher (175 vs 128 cells/high power field) in animals with lower Sat PC levels (Figure 3D-F). MCP-1 protein was localized to similar regions, but with more variation within each group (Figure 3G-I). MCP-1 protein levels in the BAL were increased 5 fold in animals with Sat PC less than 5 μmol/kg (Table 2).

## Discussion

Using a premature sheep model, we mimicked high tidal volume resuscitation with subsequent ventilation to test if several inhibitors of pro-inflammatory mediators would decrease injury. The preterm lambs had lung inflammation to 15 minutes of escalating V_T _ventilation and the subsequent ventilation. Although the study was designed to cause lung injury, the current preterm lambs had a similar magnitude increase in inflammatory markers to lambs ventilated with a tidal volume of 8 mL/kg and PEEP of 5 cmH_2_0 [14]. We did not find differences in respiratory physiology or markers of injury between the ventilation only animals and those treated with inhibitors of IL-1, IL-8, or NF-κB, but there was variable injury within each treatment group. We took advantage of this variable injury to demonstrate that the amount of Sat PC in the BALF at autopsy was associated with the amount of lung inflammation and injury. There appears to be a critical surfactant threshold (approximately 5 μmol/kg in preterm lambs) for partial protection of the preterm lung from injury and inflammation caused by initiation of ventilation with high V_T_. However the surfactant pool size measured at 2 h did not correlate with the compliances of the animals during the initial 15 min ventilation injury.

In these studies, we attempted to inhibit important pro-inflammatory mediators of early lung injury response. Since many of the pro-inflammatory cytokines are transcribed after nuclear translocation of NF-κB, we used both IV and intra-tracheal parthenolide to block NF-kB activation in 7 lambs. Parthenolide is a sesquiterpene lactone derived from the plant Feverfew which may block NF-κB activity through inhibition of Iκ-B kinase activity [26]. The NF-kB system is active in preterm sheep and responds to intra-amniotic LPS [27]. Mice also have NF-κB activity by mid-gestation which increases near the end of gestation and in early post-natal life [28]. Mice ventilated with large V_T _had NF-κB activation and cytokine production similar to mice exposed to LPS, but the cytokine increases were blocked by pretreatment with dexamthasone [29]. Our previous attempts to block lung inflammation with high dose dexamethasone or hydrocortisone were unsuccessful in the setting of initiation of ventilation in preterm sheep [2]. In a newborn piglet model of RDS, animals receiving a NF-κB inhibitor had no improvement over controls [30]. Inhibition of NF-κB in mice exposed to high V_T _ventilation and hyperoxia blocked the additive effect of hyperoxia on volutrauma suggesting differing roles of NF-κB in stretch injury verses oxidative injury [31]. In these three animal models of acute respiratory failure and in these preterm lambs, blockade of NF-κB was unable to prevent inflammation from mechanical ventilation.

We also attempted to block two of the major pro-inflammatory cytokines that are increased with high V_T _ventilation. We used recombinant human IL-1ra (Anikinra) to block IL-1 signaling. rhIL-RA is used clinically for treatment of a variety of inflammatory diseases and mutations ofIL-1ra result in severe systemic inflammation in early childhood [32]. We previously demonstrated that rhIL-1ra completely blocked lung inflammation from recombinant sheep IL-1 and decreased, but did not eliminate, the inflammatory response to LPS [16]. When adjusted for the amount of Sat PC in BAL, the IL-1 receptor blocker did not decrease lung injury in these lambs. We also tested nicotinanilide thioglycolate methyl ester (NTME), a CXCR2 inhibitor, to block IL-8 signaling. We previously demonstrated that NTME blocked recombinant ovine IL-8, but did not inhibitLPS induced lung inflammation in preterm sheep [15]. We found no decrease in ventilation mediated lung injury for this IL-8 inhibitor. Although drug levels were not measured in this study, we previously measured bio-availability of Anikinra and NTME in sheep [15,16]. Based on our previous results, the dosage used in the present study should have blocked IL-1 and IL-8 signaling. These studies, in combination with our previous study with postnatal corticosteroid treatment, suggest that blockade of pro-inflammatory responses to the initiation of ventilation of preterm infants will not be accomplished easily.

Previous preterm animal studies demonstrated improved ventilation with increasing endogenous surfactant pool sizes [7,19]. Base on the observation that animals with lower Sat PC levels had exponentially higher indicators of lung injury, we stratified the animals based on Sat PC level. We found animals with Sat PC levels less than < 5 μmol/kg (about 8 mg/kg surfactant based on assumption that Sat PC is 50% of the surfactant lipid pool) had significantly more injury than those with more Sat PC. These findings were similar to our previous findings that lambs with less than 1.9 μmol/kg Sat PC (3 mg/kg surfactant) had high PaCO_2 _on CPAP, whereas lambs with more Sat PC had normal PaCO_2 _levels [19]. The average surfactant pool of the term newborn sheep is approximately 100 mg/kg in BALF [25]. The average surfactant pool in group of animals with > 5 μmol/kg was about 15% of the levels reported at term. The average Sat PC pool for the lambs with < 5 μmol/kg was 2.5 μmol/kg, about 1.3 fold higher than that for lambs that previously survived on CPAP [19]. This lower effective pool size for the lambs on CPAP may result from the lack of intentional lung injury in those animals. The high V_T _and resultant lung injury should cause inhibition of endogenous surfactant pools [7]. In 1970, Adams found surfactant pools less than 5 mg/kg in infants who died with RDS without mechanical ventilation [33]. These lambs were date-mated and the majority of lambs (25/28) were 133 days gestation, suggesting that small variations in lung maturation at the same gestational age can affect lung injury. Small changes in endogenous surfactant levels may have larger effects on lung mechanics than larger surfactant pool size increases with surfactant treatment [7].

Small increases in the endogenous surfactant pool size could decrease the heterogeneity of lung expansion and cause a more even distribution of V_T _across the regions of the lung, and thus decrease focal injury [34]. Surfactant decreases surface tension and maintains FRC [35]. Since no PEEP was used during the first 15 min, the lambs with higher Sat PC may have had reduced regions of airway collapse. Newly secreted surfactant following birth is the large aggregate surfactant that has the best functional characteristics. High tidal volume ventilation can convert surfactant from surface-active large-aggregates to less surface-active surfactant forms [36] and these changes in surfactant forms proceed physiologic changes during ventilation [37]. The lambs with less surfactant in BAL may have had less functional surfactant and more surfactant inhibition, although these variables were not measured [38]. The induction of HSP70 in the smooth muscle of the airways was not changed by the amount of Sat PC, demonstrating an airway injury response with ventilation in all groups [1]. Our current analysis of injury based on surfactant pool size stresses the importance of the endogenous surfactant pool size on lung injury induced by the initiation of ventilation at birth.

A limitation of our study is the sample size (n = 7 to 8) for each intervention group. The study is thus powered to demonstrate only large differences between inhibitor groups and small differences from the inhibitors would not be detected. This variability in injury response is a limitation to studies with large animals. Another limitation of the study is the use of Sat PC levels after 2 hours of ventilation as a marker for endogenous surfactant pool size at birth. Surfactant is secreted into the airspace with the initiation of ventilation [9], such the pool size during the 15 min of tidal volume escalation would be smaller than the values measured at 2 hr. A final limitation of large animal studies is the difficulty in proving causality. We simply can correlate the results that lambs with increased surfactant pool sizes had decreased injury, as was seen with premature rabbits [13].

## Conclusions

Small changes in the surfactant pool size correlated with large differences in lung injury and inflammation. All the preterm lambs were surfactant deficient and had ventilator induced lung injury, but a Sat PC level of > 5 μmol/kg was sufficient to reduce the injury. Stretch injury to preterm sheep lung activates multiple, over-lapping acute phase response pathways, with cytokine production and lung inflammation as potential adverse outcomes. The lung inflammation from mechanical ventilation will likely not be prevented by the inhibition of any particular pro-inflammatory cytokine or by a more global inhibition with postnatal steroids [2]. While surfactant pools may serve as a biomarker for eventual lung injury from mechanical ventilation, there is no practical way to measure the pool size prior to or shortly after resuscitation in infants. Minor changes in surfactant pool sizes, as demonstrated by this study, are important for lung injury and support the use of antenatal steroids to increase surfactant.

## Abbreviations

BALF: Bronchoalveolar lavage fluid; BPD: Bronchopulmonary dysplasia; Egr-1: Early growth response protein 1; HSP70: Heat Shock protein 70; MCP-1: Monocyte chemotactic protein 1; NF-κB: Nuclear factor kappa B; PEEP: Positive End Expiratory Pressure; PIP: Peak Inspiratory Pressure; Sat PC: Saturated phosphatidylcholine; VEI: Ventilator Efficiency Index; V_T _:Tidal Volume

## Competing interests

The authors declare they have no competing interests to declare.

## Authors' contributions

NHH did the animals studies, the molecular analysis, statistical analysis and drafted the manuscript. SGK did research design, molecular analysis and manuscript development. JJP did animal care and manuscript development. IN and GRP did animal breeding and manuscript development. MI did saturation PC analysis and manuscript editing. AHJ conceived the study, participated in its design, and help draft the manuscript. All authors have read and approve the manuscript.

## References

[B1] Hillman NH Kallapur SG Pillow JJ Moss TJ Polglase GR Nitsos I Jobe AH Airway injury from initiating ventilation in preterm sheep Pediatr Res 2009 67 1 60 65 10.1203/PDR.0b013e3181c1b09e PMC2795027 19816239

[B2] Hillman NH Pillow JJ Ball MK Polglase GR Kallapur SG Jobe AH Antenatal and postnatal corticosteroid and resuscitation induced lung injury in preterm sheep Respiratory Research 2009 10 124 10.1186/1465-9921-10-124 20003512 PMC2802354

[B3] Kotecha S Chan B Azam N Silverman M Shaw RJ Increase in interleukin-8 and soluble intercellular adhesion molecule-1 in bronchoalveolar lavage fluid from premature infants who develop chronic lung disease Archives of disease in childhood 1995 72 2 F90 96 7712280 10.1136/fn.72.2.f90 PMC2528395

[B4] Kotecha S Wilson L Wangoo A Silverman M Shaw RJ Increase in interleukin (IL)-1β and IL-6 in bronchoalveolar lavage fluid obtained from infants with chronic lung disease of prematurity Pediatr Res 1996 40 2 250 256 10.1203/00006450-199608000-00010 8827773

[B5] Baier RJ Majid A Parupia H Loggins J Kruger TE CC chemokine concentrations increase in respiratory distress syndrome and correlate with development of bronchopulmonary dysplasia Pediatric Pulmonology 2004 37 2 137 148 10.1002/ppul.10417 14730659

[B6] Bohrer B Silveira RC Neto EC Procianoy RS Mechanical ventilation of newborns infant changes in plasma pro- and anti-inflammatory cytokines The Journal of Pediatrics 156 1 16 19 10.1016/j.jpeds.2009.07.027 19783005

[B7] Ikegami M Jobe AH Yamada T Seidner S Relationship between alveolar saturated phosphatidylcholine pool sizes and compliance of preterm rabbit lungs. The effect of maternal corticosteroid treatment The American Review of Respiratory Disease 1989 139 2 367 369 2913885 10.1164/ajrccm/139.2.367

[B8] Wada K Jobe AH Ikegami M Tidal volume effects on surfactant treatment responses with the initiation of ventilation in preterm lambs J Appl Physiol 1997 83 4 1054 1061 9338410 10.1152/jappl.1997.83.4.1054

[B9] Jacobs H Jobe A Ikegami M Jones S Accumulation of alveolar surfactant following delivery and ventilation of premature lambs Exp Lung Res 1985 8 2-3 125 140 10.3109/01902148509057517 3839748

[B10] Wallace MJ Probyn ME Zahra VA Crossley K Cole TJ Davis PG Morley CJ Hooper SB Early biomarkers and potential mediators of ventilation-induced lung injury in very preterm lambs Respiratory Research 2009 10 19 10.1186/1465-9921-10-19 19284536 PMC2662809

[B11] Morley CJ Davis PG Doyle LW Brion LP Hascoet JM Carlin JB Nasal CPAP or intubation at birth for very preterm infants The New England Journal of Medicine 2008 358 7 700 708 10.1056/NEJMoa072788 18272893

[B12] Ammari A Suri MS Milisavljevic V Sahni R Bateman DA Sanocka U Ruzal-Shapiro C Wung JT Polin RA Variables associated with the early failure of nasal CPAP in very low birth weight infants Journal of Pediatrics 2005 147 341 347 10.1016/j.jpeds.2005.04.062 16182673

[B13] Jobe AH Ikegami M Jacobs HC Jones SJ Surfactant pool sizes and severity of respiratory distress syndrome in prematurely delivered lambs The American review of respiratory disease 1983 127 6 751 755 6552894 10.1164/arrd.1983.127.6.751

[B14] Polglase GR Hillman NH Pillow JJ Cheah FC Nitsos I Moss TJ Kramer BW Ikegami M Kallapur SG Jobe AH Positive end-expiratory pressure and tidal volume during initial ventilation of preterm lambs Pediatr Res 2008 64 5 517 522 10.1203/PDR.0b013e3181841363 18596572 PMC2637939

[B15] Kallapur SG Moss TJ Auten RL Nitsos I Pillow JJ Kramer BW Maeda DY Newnham JP Ikegami M Jobe AH IL-8 signaling does not mediate intra-amniotic LPS-induced inflammation and maturation in preterm fetal lamb lung Am J Physiol 2009 297 3 L512 519 10.1152/ajplung.00105.2009 PMC2739771 19574422

[B16] Kallapur SG Nitsos I Moss TJ Polglase GR Pillow JJ Cheah FC Kramer BW Newnham JP Ikegami M Jobe AH IL-1 mediates pulmonary and systemic inflammatory responses to chorioamnionitis induced by lipopolysaccharide American journal of respiratory and critical care medicine 2009 179 10 955 961 10.1164/rccm.200811-1728OC 19234101 PMC2684020

[B17] Sheehan M Wong HR Hake PW Malhotra V O'Connor M Zingarelli B Parthenolide, an inhibitor of the nuclear factor-kappaB pathway, ameliorates cardiovascular derangement and outcome in endotoxic shock in rodents Mol Pharmacol 2002 61 5 953 963 10.1124/mol.61.5.953 11961112

[B18] Lowry OH Rosebrough NJ Farr AL Randall RJ Protein measurement with the Folin phenol reagent J Biol Chem 1951 193 1 265 275 14907713

[B19] Mulrooney N Champion Z Moss TJ Nitsos I Ikegami M Jobe AH Surfactant and Physiological Responses of Preterm Lambs to Continuous Positive Airway Pressure American journal of respiratory and critical care medicine 2005 171 1 6 10.1164/rccm.200406-774OC 15502113

[B20] Kallapur SG Willet KE Jobe AH Ikegami M Bachurski C Intra-amniotic endotoxin: Chorioamnionitis precedes lung maturation in preterm lambs Am J Physiol 2001 280 L527 L536 10.1152/ajplung.2001.280.3.L527 11159037

[B21] Kallapur SG Willet KE Jobe AH Ikegami M Bachurski CJ Intra-amniotic endotoxin: chorioamnionitis precedes lung maturation in preterm lambs Am J Physiol Lung Cell Mol Physiol 2001 280 3 L527 536 11159037 10.1152/ajplung.2001.280.3.L527

[B22] Hillman NH Moss TJ Nitsos I Kramer BW Bachurski CJ Ikegami M Jobe AH Kallapur SG Toll-like receptors and agonist responses in the developing fetal sheep lung Pediatr Res 2008 63 4 388 393 10.1203/PDR.0b013e3181647b3a 18356744

[B23] Kramer BW Moss TJ Willet KE Newnham JP Sly PD Kallapur SG Ikegami M Jobe AH Dose and time response after intraamniotic endotoxin in preterm lambs Am J Respir Crit Care Med 2001 164 6 982 988 11587983 10.1164/ajrccm.164.6.2103061

[B24] Shah TAHN Nitsos I Polglase GR Pillow JJ Newnham JP Jobe AH Kallapur SG Pulmonary and Systemic Expression of Monocyte Chemotactic Proteins in Preterm Sheep Fetuses Exposed to LPS Induced Chorioamnionitis Pediatr Res 2010 68 3 210 5 10.1203/PDR.0b013e3181e9c556 20703142 PMC3123719

[B25] Glatz T Ikegami M Jobe A Metabolism of exogenously administered natural surfactant in the newborn lamb Pediatr Res 1982 16 711 715 10.1203/00006450-198209000-00002 6897111

[B26] Saadane A Masters S DiDonato J Li J Berger M Parthenolide inhibits IkappaB kinase, NF-kappaB activation, and inflammatory response in cystic fibrosis cells and mice American journal of respiratory cell and molecular biology 2007 36 6 728 736 10.1165/rcmb.2006-0323OC 17272824 PMC1899341

[B27] Cheah FC Pillow JJ Kramer BW Polglase GR Nitsos I Newnham JP Jobe AH Kallapur SG Airway inflammatory cell responses to intra-amniotic lipopolysaccharide in a sheep model of chorioamnionitis Am J Physiol 2009 296 3 L384 393 10.1152/ajplung.90547.2008 PMC2660220 19118089

[B28] Dohlen G Odland HH Carlsen H Blomhoff R Thaulow E Saugstad OD Antioxidant activity in the newborn brain: a luciferase mouse model Neonatology 2008 93 2 125 131 10.1159/000107777 17785990

[B29] Held HD Boettcher S Hamann L Uhlig S Ventilation-induced chemokine and cytokine release is associated with activation of nuclear factor-kappaB and is blocked by steroids American journal of respiratory and critical care medicine 2001 163 3 Pt 1 711 716 11254529 10.1164/ajrccm.163.3.2003001

[B30] von Bismarck P Klemm K Garcia Wistadt CF Winoto-Morbach S Schutze S Krause MF Selective NF-kappaB inhibition, but not dexamethasone, decreases acute lung injury in a newborn piglet airway inflammation model Pulmonary pharmacology & therapeutics 2009 22 4 297 304 10.1016/j.pupt.2009.02.002 19254776

[B31] Liu YY Liao SK Huang CC Tsai YH Quinn DA Li LF Role for nuclear factor-kappaB in augmented lung injury because of interaction between hyperoxia and high stretch ventilation Transl Res 2009 154 5 228 240 10.1016/j.trsl.2009.06.006 19840764

[B32] Aksentijevich I Masters SL Ferguson PJ Dancey P Frenkel J van Royen-Kerkhoff A Laxer R Tedgard U Cowen EW Pham TH An autoinflammatory disease with deficiency of the interleukin-1-receptor antagonist The New England Journal of Medicine 2009 360 23 2426 2437 10.1056/NEJMoa0807865 19494218 PMC2876877

[B33] Adams FH Fujiwara T Emmanouilides GC Raiha N Lung phospholipid of the human fetus and infants with and without hyaline membrane disease Journal of Pediatrics 1970 77 833 10.1016/S0022-3476(70)80244-X 5504075

[B34] Jobe AH Hillman N Polglase G Kramer BW Kallapur S Pillow J Injury and inflammation from resuscitation of the preterm infant Neonatology 2008 94 3 190 196 10.1159/000143721 18832854

[B35] Scarpelli EM Mautone AJ DeFouw DO Clutario BC Intraalveolar bubbles and bubble films: II. Formation in vivo through adulthood The Anatomical record 1996 246 2 245 270 10.1002/(SICI)1097-0185(199610)246:2<245::AID-AR12>3.0.CO;2-O 8888967

[B36] Veldhuizen RAW Marcou J Yao LJ Alveolar surfactant aggregate conversion in ventilated normal and injured rabbits Am J Physiol 1996 14 152 158 10.1152/ajplung.1996.270.1.L152 8772538

[B37] Maruscak AA Vockeroth DW Girardi B Sheikh T Possmayer F Lewis JF Veldhuizen RA Alterations to surfactant precede physiological deterioration during high tidal volume ventilation Am J Physiol 2008 294 5 L974 983 10.1152/ajplung.00528.2007 18344412

[B38] Michna J Jobe AH Ikegami M Positive end-expiratory pressure preserves surfactant function in preterm lambs American journal of respiratory and critical care medicine 1999 160 2 634 639 10430740 10.1164/ajrccm.160.2.9902016

